# Endogenous Hormones Inhibit Differentiation of Young Ears in Maize (*Zea mays L.*) Under Heat Stress

**DOI:** 10.3389/fpls.2020.533046

**Published:** 2020-10-22

**Authors:** Hui-Qin Wang, Peng Liu, Ji-Wang Zhang, Bin Zhao, Bai-Zhao Ren

**Affiliations:** State Key Laboratory of Crop Biology, Agronomy College of Shandong Agricultural University, Tai’an, China

**Keywords:** high temperature, ear development, transcriptomic, floret, hormones

## Abstract

Global warming frequently leads to extreme temperatures, which pose a serious threat to the growth, development, and yield formation of crops such as maize. This study aimed to deeply explore the molecular mechanisms of young ear development under heat stress. We selected the heat-tolerant maize variety Zhengdan 958 (T) and heat-sensitive maize variety Xianyu 335 (S), and subjected them to heat stress in the V9 (9th leaf), V12 (12th leaf), and VT (tasseling) growth stages. We combined analysis of the maize phenotype with omics technology and physiological indicators to compare the differences in young ear morphology, total number of florets, floret fertilization rate, grain abortion rate, number of grains, and main metabolic pathways between plants subjected to heat stress and those left to develop normally. The results showed that after heat stress, the length and diameter of young ears, total number of florets, floret fertilization rate, and number of grains all decreased significantly, whereas the length of the undeveloped part at the top of the ear and grain abortion rate increased significantly. In addition, the differentially expressed genes (DEGs) in young ears were significantly enriched in the hormone signaling pathways. The endogenous hormone content in young ears exhibited different changes: zeatin (ZT) and zeatin riboside (ZR) decreased significantly, but gibberellin acid_3_ (GA_3_), gibberellin acid_4_ (GA_4_), and abscisic acid (ABA) increased significantly, in ears subjected to heat stress. In the heat-tolerant maize variety, the salicylic acid (SA), and jasmonic acid (JA) content in the vegetative growth stage also increased in ears subjected to heat stress, whereas the opposite effect was observed for the heat-sensitive variety. The changes in endogenous hormone content of young ears that were subjected to heat stress significantly affected ear development, resulting in a reduction in the number of differentiated florets, fertilized florets and grains, which ultimately reduced the maize yield.

## Introduction

In many parts of the world, temperatures hit record highs in 2019. Concentrations of greenhouse gases are rising, making 2015–2019 the warmest 5 years on record. In 2016, the global surface temperature reached a new high of 14.84°C and global temperature records were broken for three consecutive years (Sun et al., 2017). The “greenhouse effect” is becoming more intense, and rising temperatures have become a global problem affecting crop growth and yield. In recent years, frequent extreme weather and meteorological disasters have become the main factors restricting crop production (Högy et al., 2019). It has been predicted that the global temperature will increase by 1.5–6°C in the future (Müller et al., 2017), causing the expectation that heat stress will become the main abiotic stress factor for crop production in times to come. Previous research has shown that heat stress has a major impact on crop yield: for every 1°C increase in global temperature over the appropriate temperature needed to grow crops, the crop yield decreases by 2.5–16% (Battisti and Naylor, 2009). According to a USDA report, the world produced 10.34 and 11.23 million tons of maize in 2017 and 2018, while global maize consumption was 10.62 and 11.34 million tons, respectively. In 2022, the global maize consumption is expected to exceed 13 million tons. Furthermore, the FAO estimates that by 2050 the world will need 70–110% more food than today for meeting the projected population growth, and this increase would need to be realized largely by improving the efficiency of the production process without increasing the finite area of land currently assigned to agriculture (Ravic and Sandy, 2016). Due to the increasing gap between the supply and demand for maize, maintaining the total maize yield is a priority. This requires clarification of the molecular mechanisms involved in responses of maize to heat stress, improving its thermotolerance, and increasing the yield per unit area.

Maize is very sensitive to heat stress, especially during the critical stage when young ears differentiate. Observations show heat stress responses including reductions in the growth rate of young ears, duration of ear differentiation, and number of floret differentiations leading to anomalies such as reduced ear size and increased floret abortion, and eventually, to a significant reduction in the number of grains per spike (Cicchino et al., 2010; Hossain and Teixeira, 2012; Sobol et al., 2014). Of all the stages in the life cycle of maize, anthesis shows the highest sensitivity to heat stress. The optimum temperature for tasseling is 26–27°C, and exposure to temperatures above 30°C results in a drop in production (Lobell et al., 2011). The main reasons for production loss due to heat stress are a reduced number of filaments, delayed silking time, and decreased silking rate and filament viability. These changes in ear development ultimately result in reduced florescence, insufficient pollination and fertilization, and significantly decreased grain numbers (Wu C. et al., 2019). For rice, the most damaging heat stress responses occur at either the anthesis stage or the gametogenesis stage and include inhibited pollen viability and pollen tube growth, which result in a higher spikelet sterility rate (Jagadish et al., 2010, 2013). For heat-sensitive rice varieties, the main reason for decreases in grain yield is a decrease in spikelet fertility (Wu et al., 2016). In general, the harm done by heat stress during the reproductive growth stage is much more severe than that done during the vegetative growth stage, and the harm done during the reproductive organ differentiation stage is irreversible (Obata et al., 2015).

About 18% of the gene expression of *Sorghum bicolor* is altered in response to heat stress (Johnson et al., 2014). In heat-tolerant genotypes, gene expression is mainly up-regulated, but in heat-sensitive genotypes it is mainly down-regulated (Craita et al., 2011). Over half of the differentially expressed genes (DEGs) are involved in endoplasmic reticulum protein processing, metabolic pathways, and biosynthesis of secondary metabolites (Liu et al., 2014). Transcriptome data shows that there is a broad interaction between plant thermotolerance and cytokinin signaling (Černý et al., 2014). Understanding the dynamic changes in gene expression will be helpful in revealing the signaling pathways involved in the response of young ears to heat stress. However, relatively few transcriptome studies, which consider different heat-resistant maize varieties, technology have been carried out, using RNA sequencing (RNA-seq), on ear responses to heat stress during multiple growth stages. In this study, we used RNA-seq based on the Illumina sequencing platform for the transcriptome analysis of ears subjected to heat stress. We aimed to identify the molecular mechanisms carrying the signals involved in heat stress responses during the development of young ears at various growth stages for different heat-resistant maize varieties. The results will contribute to the research data in the field.

Hormones, which play an important role in plant development, are capable of regulating various life processes efficiently, even at extremely low concentrations. When external stress disrupts a plant’s homeostasis, hormones enable plants to perceive these unfavorable changes in the external environment and counter them to maintain homeostasis (Wu et al., 2018). A single hormone might regulate many physiological processes, and a single physiological process might need the synergistic action of many hormones. This gives rise to a complex regulatory network of hormones that collectively regulate life processes such as differentiation and grain development in young ears (Wolters and Jürgens, 2009). For example, cytokinins can regulate the ear size and thermotolerance of rice (Wu et al., 2017). A decrease in cytokinin content, induced by heat stress, has been shown to lead to inhibited ear differentiation, floret fertility, pollen viability, and anther cracking, as well as a smaller grain size, none of which are conducive to yield formation (Sobol et al., 2014; Wu et al., 2016). Heat stress causes the abscisic acid (ABA) content in anthers and grains to increase and gibberellin content to decrease, which in turn leads to floret abortion. Spraying cytokinin can protect the floret primordia, allowing them to develop normally (Sobol et al., 2014). A high gibberellin acid_3_ content in young panicles is conducive to the differentiation of spikelets in wheat, and a high content of zeatin promotes the development of florets, but a high ABA content during the booting stage inhibits the development of florets (Sheng et al., 2001). Spraying brassinolide during panicle differentiation can increase the number of spikelets, which ultimately increases the number of grains per panicle (Chen et al., 2019). Spraying cytokinins can also mitigate heat-stress-related damage to heat-sensitive rice varieties (Wu et al., 2016). Salicylic acid (SA) and jasmonic acid (JA) are considered to be important signaling substances with respect to plant stress responses, especially those responses that improve the thermotolerance of a crop (Clarke et al., 2009; Lu et al., 2009; Keykha et al., 2014). The content of endogenous SA and JA in plants increases under heat stress (Escandón et al., 2016; Scalabrin et al., 2016). Nevertheless, the relationship between the changes in endogenous hormone content and development of young ears in maize under heat stress is still unclear. To fill this gap, our study aimed to systematically integrate the phenotype, physiology, and omics of maize under heat stress.

Previous studies on maize responses to heat stress mainly focused on ear traits, yield, ZR, GA, and ABA content in a single growth stage. The missing element is a comprehensive analysis of the effects of heat stress response of maize on young ear development, omics, and endogenous hormones, in different heat-resistant maize varieties, during different growth stages. The current study combined the analysis of phenotypical and physiological changes with transcriptome analysis, and deeply explored the molecular mechanism of ear development under heat stress, providing strong scientific and technological support for mitigating crop disasters, stabilizing yields, improving thermotolerance, and breeding stress-resistant maize varieties.

## Materials and Methods

### Plant Materials and Growth Condition

The experiments were carried out during the summer maize growing season in 2018 and 2019 at Shandong Agricultural University Farm, Tai’an, SD, China (36° 09′ N, 117° 09′ E). After applying a screening process, we selected and planted two hybrid varieties of summer maize with different heat tolerance capacities: Zhengdan 958 (T), which represents the heat-tolerant variety, and Xianyu 335 (S), which represents the heat-sensitive variety. Pot experiments were set, and a basal fertilizer of 4.80 *g* N, 1.92 *g* P_2_O_5_, and 3.84 *g* K_2_O was applied per pot before sowing. All plants were sown in plastic pots (40 cm length × 40 cm width × 50 cm depth). To ensure that moisture, diseases, pests and weeds did not restrict plant growth, additional management measures developed for high-yield cultivation were adopted.

### Heat Stress Treatment and Sampling

**FIGURE 1:**
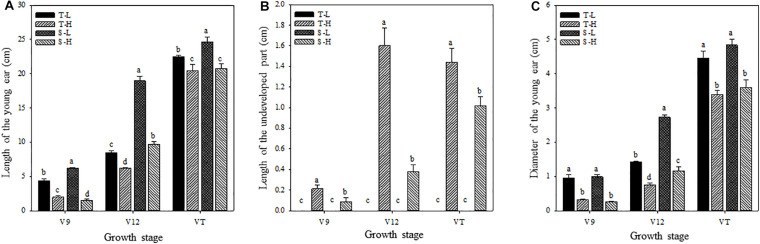
Effects of heat stress on the length of the young ear **(A)**, the length of the undeveloped part at the top of the ear **(B)**, and the ear diameter **(C)**. T and S represent the heat-tolerant variety and the heat-sensitive variety, respectively. L represents the natural environment and H represents the heat-stressed environment. V9, V12, and VT represent the 9^th^ leaf, 12^th^ leaf, and tasseling stages. Vertical bars represent ± SD of the mean. a, b, c, and d indicate significant difference among different treatments (*n* = 3, *P* < 0.05).

While not being subjected to heat stress, the plants were left to grow in a natural environment, in this case the ambient environment at the research station. When the plants entered the V9 (9th leaf), V12 (12th leaf), or VT (tasseling) stage in their development, all plants in the test groups that were required to be subjected to heat stress during that stage were moved to transparent plastic sheds (12 m length × 6 m width, with a light transmittance of 92%), which featured automatic electric heaters used to control the temperature. Inside the sheds, the test groups were exposed to a heat-stressed environment with a day-time temperature of 40°C and night-time temperature of 30°C. The sheds had 60 cm air vents on all sides to ensure proper ventilation and establish identical concentrations of carbon dioxide and air humidity on the inside and outside. The temperature and humidity were recorded using a thermohygrograph, GSP-6 (Jingchuang Instruments Co., Ltd; Figure 1). Plants that were grown only in a natural environment were considered the controls (L), the experiment included 12 treatments: T-H-V9, T-L-V9, T-H-V12, T-L-V12, T-H-VT, T-L-VT, S-H-V9, S-L-V9, S-H-V12, S-L-V12, S-H-VT, and S-L-VT, where T and S denote the heat-tolerant maize variety Zhengdan 958 and heat-sensitive maize variety Xianyu 335, respectively, H denotes a heat stress group, L denotes a control group, and V9, V12, and VT denote the growth stages during which the plants in the corresponding heat stress test group were subjected to heat stress.

After subjecting the plants in the relevant test groups to heat stress for 10 days, we collected 10 young ears from plants in each group and immediately froze them in liquid nitrogen, then stored them at −80°C for quantifying endogenous hormones and RNA-seq analysis, each group had three replicates. We also collected 10 young ears that were used to characterize the ear morphology, as described in the next section. After completing the sampling process, the remaining plants in the test groups were removed from the shed and returned to the natural environment, where they were left to grow naturally until harvest time.

### Ear Characterization

To analyze the 10 young ears collected for the purpose of ear characterization, we measured the length of the ear, the length of the undeveloped part at the top of the ear, and the ear diameter with a ruler.

### Floret Differentiation

Before the silking stage set in, we selected 15 representative plants in each treatment for labeling, according to the method of Cui et al. (2014). After pollination finished, we peeled the bracts from the ear and counted the total number of differentiated florets. The count was divided into three parts: first, after shaking gently, we counted the number of filaments that were shed and the number of filaments that were not shed but were wilted at the base and added these counts to obtain the number of fertilized florets; second, the number of fresh filaments that were not shed was taken to represent the number of unfertilized florets; and third, the number of filaments that were not drawn out was taken to represent the number of degenerated florets. We then calculated the floret fertilization rate and grain abortion rate, using the following formulae:

Floret fertilization rate (%) = (number of fertilized florets/number of total florets) × 100

Grain abortion rate (%) = (number of fertilized florets – grain numbers) × 100/number of fertilized florets

### RNA Extraction and Sequencing

Total RNA was isolated from the ears using a TRIzol reagent according to manufacturer’s instructions (Invitrogen). An additional DNase I digestion step was performed to ensure that the samples were not contaminated with genomic DNA. RNA purity was assessed using the Nanodrop-2000. Each RNA sample had an A260:A280 ratio above 1.8 and A260:A230 ratio above 2.

The methods for library preparation and sequencing were modified from those described by Garzón-Martínez et al. (2012). The paired-end (PE) RNA-seq libraries were prepared following Illumina’s protocols and sequenced on the Illumina HiSeq^TM^ X Ten platform. Data analysis and base calling were performed using the Illumina instrument software. High throughput sequencing was performed at Ori-Gene Science and Technology Co., Ltd. (Beijing, China).

### RNA-seq Quality Pre-processing and Analysis Results

The RNA-seq results were qualified by the Q20 standard, which showed that the sequencing quality was acceptable. Raw reads from sequencing machines contain dirty reads that include adapters, as well as unknown or low-quality bases, which would negatively affect any bioinformatics analysis. Therefore, dirty raw reads (i.e., reads with adapters, reads with an unknown nucleotides ratio larger than 5%, and low-quality reads) were discarded. After filtering the raw reads, *de novo* assembly of the transcriptome was carried out with Trinity, a short reads assembly program. Trinity connected the contigs and obtained sequences defined as unigenes. The generated unigenes were used for BLASTX and annotation against a protein database, such as the KEGG database, with a cut-off *E*-value of 0.00001. KEGG is a major public pathway-related database that can analyze a gene product during metabolic processes and related gene functions in cellular processes. With the help of the KEGG database, we could further study the complex biological behaviors of genes, and through KEGG annotation we could obtain pathway annotation for unigenes. KEGG pathway annotation was performed using the Blastall software against the KEGG database.

### Quantitative Real Time (qRT)-PCR Analysis

The quantitative real time (qRT-PCR) analysis was carried out for the further verification of RNA–Seq data using gene–specific primer sets (Supplementary Table 1). The cDNA was synthesized with a Goldenstar^TM^ RT6 cDNA Synthesis Kit Ver 2 (TsingKe Biotech Co., Ltd, Beijing, China). Using LineGene 9600 Plus (Bioer, Hangzhou, China) and SYBR Green I Mixture (TsingKe) for amplification, detection, and data analysis. The specific primers used for qRT-PCR were designed from National Center for Biotechnology Information (NCBI) and those were synthesized by TsingKe Biotech Co., Ltd (Beijing, China). GAPDH (Zm00001d049641) was used as the reference gene (Magneschi et al., 2009). The fluorescence was measured at the end of each cycle for quantification, and three technical replicates were performed. Relative expression levels were calculated using the 2^–ΔΔCt^ method.

### Availability of Supporting Data

All sequences were deposited in the NCBI and can be accessed in the Short Read Archive (SRA) under accession number: SRP250388.

### Extraction and Determination of Endogenous Hormones

The methods for extraction and purification of ABA, JA, SA, ZR, ZT, GA_3_, and GA_4_ were modified from those described by Engelberth et al. (2003). Endogenous hormones were analyzed using a triple quadrupole mass spectrometer (ACQUITY UPLC I-Class/Xevo TQ-S, Waters, Milford, MA, United States) equipped with an electrospray ion source (ESI). The LC separation was performed on a reversed-phase C18 column (ACQUITY UPLC BEH, 1.7 μm, 2.1 mm × 100.0 mm, Waters, Milford, MA, United States) using a binary solvent system composed of water with 0.1% acetonitrile, 0.1% formic acid (mobile phase A), and MeOH (mobile phase B) at a flow rate of 0.4 mL min^–1^. Standard ABA, JA, ZR, ZT, GA_3_, GA_4_, and isotopically labeled internal standards including D-JA, D-ABA, D-GA_4_, and D-ZT were purchased from OlChemIm Ltd. (OlChemIm Ltd., Olomouc, Czech Republic), and standard SA was purchased from Sigma. Methanol, acetonitrile, and formic acid were purchased from Fisher (Thermo Fisher Scientific, Waltham, MA, United States).

### Statistical Analysis

Statistical analyses were performed using the analysis of variance (ANOVA) algorithm from the General Linear Model procedure of the SPSS software, version 26.0 (SPSS Inc). Unless indicated otherwise, significant differences among different plants were given at *p* ≤ 0.05. The values of the mean, among treatments, were compared using the Tukey Honestly Significant Difference test. Figures were plotted using the SigmaPlot 14.0 program.

## Results

### Characteristics of Young Maize Ears

**FIGURE 2:**
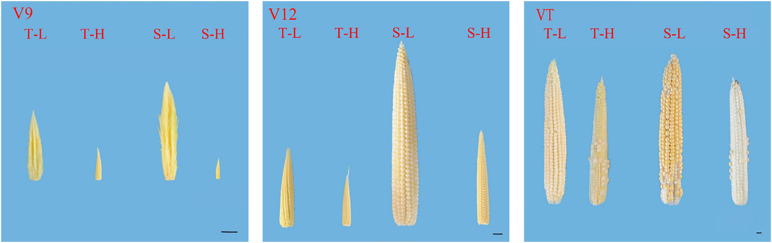
Effects of heat stress on the morphology of young ears of two varieties of summer maize with a different capacity for heat tolerance. T and S represent the heat-tolerant variety and the heat-sensitive variety, respectively. L represents the natural environment and H represent the heat-stressed environment. V9, V12, and VT represent the 9^th^ leaf, 12^th^ leaf, and tasseling stages. The length of the ruler is 1 cm.

After being subjected to heat stress at any of the stages included in the experiment, the length and diameter of young ears was significantly reduced, and the proportion of the undeveloped part at the top of the ear was significantly increased. The average ear length for the heat-tolerant maize variety after heat stress application during the V9, V12, and VT stage was 54.44, 26.54, and 8.89% lower than that of the control groups, respectively, and for the heat-sensitive maize variety, it was 75.97, 48.89, and 15.6% lower. For the ear diameter, the heat-tolerant maize variety was 66.39, 46.49, and 23.63% lower than that of the control in the V9, V12, and VT stage, respectively, and the heat-sensitive maize variety was 74.44, 57.25, and 25.62% lower (Figure 1). In addition, the growth stage of the ear was delayed, and during the VT stage, severe abortion of the upper and base grains was observed, while morphologies of the control ears were better and their grains were fully developed (Figure 2).

### Total Number of Florets and Floret Fertilization Rate

**FIGURE 3:**
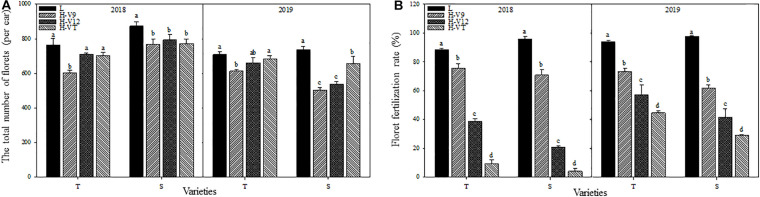
Effect of heat stress on the total number of differentiated florets in the ear **(A)** and the floret fertilization rate **(B)**. T and S represent the heat-tolerant variety and the heat-sensitive variety, respectively. L represents the natural environment and H represents the heat-stressed environment. V9, V12, and VT represent the 9^th^ leaf, 12^th^ leaf, and tasseling stages. Vertical bars represent ± SD of the mean. a, b, c, and d indicate significant difference among different treatments (*n* = 3, *P* < 0.05).

The total number of florets in young ears decreased significantly after heat stress in 2 years. 2018 for example, despite having a different capacity for heat tolerance, both maize varieties showed that the most significant decrease happened during the V9 stage, for which the decrease reached 21.07% for the heat-tolerant variety and 12.06% for the heat-sensitive variety. When heat stress was applied during the V12 or VT stage, the total number of florets showed a greater decrease in the heat-sensitive maize variety (Figure 3A). Heat stress also affected the process of floret fertilization. The floret fertilization rates of the heat-tolerant and heat-sensitive maize varieties were only 9.51% and 4.14%, respectively, after heat stress application during the VT stage, and compared with the control group, the floret fertilization rates were 89.26% and 95.68% lower. After heat stress application during the V12 stage, the floret fertilization rate in the heat-tolerant and heat-sensitive varieties was 56.38% and 78.11% lower than that for the control groups, respectively. Although the effect of heat stress application during the V9 stage, on the floret fertilization rate, was significantly less than that of heat stress application during the V12 and VT stages, the floret fertilization rate was still significantly lower than that of the control groups (Figure 3B).

### Grain Numbers and Abortion Rate

**FIGURE 4:**
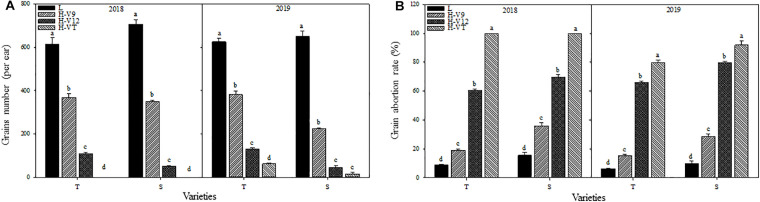
Effect of heat stress on the grains number **(A)** and grain abortion rate **(B)**. T and S represent the heat-tolerant variety and the heat-sensitive variety, respectively. L represents the natural environment and H represents the heat-stressed environment. V9, V12, and VT represent the 9^th^ leaf, 12^th^ leaf, and tasseling stages. Vertical bars represent ± SD of the mean. a, b, c, and d indicate significant difference among different treatments (*n* = 3, *P* < 0.05).

After being subjected to heat stress during any of the stages included in the experiment, the grain numbers for both varieties were significantly reduced and reduction was greater for the heat-sensitive maize variety than that for the heat-tolerant variety. Number of grains decreased the most when the heat stress occurred during the VT stage. According to the data for 2018, the grain number for the heat-tolerant maize variety after heat stress application during the V9 or V12 stage was 40.12% and 82.42% lower than that for the control groups, respectively, and for the heat-sensitive maize variety it was 50.68% and 92.89% lower (Figure 4A).

After being subjected to heat stress, the grain abortion rate for the heat-sensitive maize variety was always higher than that for the heat-tolerant maize variety. The abortion rate for both varieties was the highest after heat stress application during the VT stage, followed by the V12 stage; it was the lowest after heat stress application during the V9 stage. Indeed, in 2018, after applying heat stress during the VT stage, the grain abortion rate for both the heat-tolerant and the heat-sensitive maize variety was 100%. When heat stress was applied during the V9 or V12 stage, the grain abortion rate was 19.09% and 60.53% for the heat-tolerant variety, and 35.90% and 69.84% for the heat-sensitive variety, respectively (Figure 4B).

As shown in Table 1, the length of the young ear, the length of the undeveloped part at the top of the ear, the ear diameter and the grain abortion rate exhibited extremely significant differences between varieties, temperatures, stages during which heat stress occurred, and the interactions between various factors. As for the total number of florets, there were significant or extremely significant differences between varieties, temperatures and growth stages during which heat stress was applied, but no significant differences between the types of interactions. In addition, there was no significant difference in the floret fertilization rate between varieties, but there were significant differences between temperatures, heat stages during which heat stress occurred, and the interactions of various factors.

**TABLE 1:** ANOVA (*F* value) of the effects of heat stress applied during different growth stages on the differentiation of young ear in maize varieties with different capacities for heat tolerance.

Source of variation	Variable					
	The number of total florets	Floret fertilization rate	Grain abortion rate	Length of the young ear	Length of the undeveloped part	Diameter of the young ear
Variety	127.99**	2.19^ns^	299.53**	349.17**	153.29**	87.88**
Temperature	94.39**	6788.58**	13715.59**	663.73**	1108.97**	596.02**
Stage	4.44*	834.07**	2225.28**	4669.03**	190.33**	2643.78**
Variety × Temperature	0.05^ns^	149.90**	5.35*	139.01**	153.29**	24.35**
Variety × Stage				161.68**	47.46**	40.59**
Temperature × Stage				29.79**	190.33**	13.60**
Variety × Temperature × Stage	2.47^ns^	9.72**	30.02**	27.21**	47.46**	9.95**

*Significant at *P* < 0.05 (*n* = 3); **Significant at *P* < 0.01 (*n* = 3); and ^ns^non-significant.

### Analysis of Gene Expression and Differentially Expressed Genes

In the heat-tolerant maize variety, there were 49256, 50029, and 53197 genes expressed in the V9, V12, and VT stage, respectively. The highest number of DEGs (*p* value < 0.05 and | log_2_ fold change| ≥1), which was observed in the V9 stage, was 1489; among these genes, 680 were up-regulated, and 809 were down-regulated. In the heat-sensitive maize variety, there were 50776, 52985, and 53950 genes expressed in the V9, V12, and VT stage, respectively. The highest number of DEGs, which was observed in the V12 stage, was 2174; among these genes, 1606 were up-regulated and 568 were down-regulated (Table 2).

**TABLE 2:** Summary of genes expressed for different treatments.

Control VS Case	All_gene	All_DEG	UP	DOWN
T-L-V9 VS T-H-V9	49256	1489	680	809
T-L-V12 VS T-H-V12	50029	676	271	405
T-L-VT VS T-H-VT	53197	340	192	148
S-L-V9 VS S-H-V9	50776	61	44	17
S-L-V12 VS S-H-V12	52985	2174	1606	568
S-L-VT VS S-H-VT	53950	216	69	147

*All_gene represent the total number of genes expressed under heat stress, all_DEG represent the number of differently expressed genes, UP, and DOWN represent the number of genes that are up-regulated and down-regulated under heat stress, respectively. T and S represent the heat-tolerant variety and the heat-sensitive variety, respectively. L represents the natural environment and H represents the heat-stressed environment. V9, V12, and VT represent the 9^th^ leaf, 12^th^ leaf, and tasseling stages.*

### KEGG Pathway Analysis of DEGs

**FIGURE 5:**
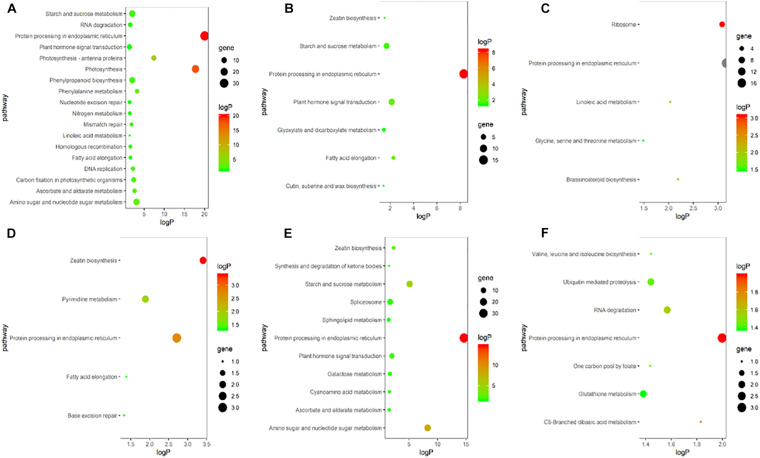
KEGG enrichment bubble chart of differentially expressed genes in ears under heat stress. **(A–C)** represent T in V9, V12, and VT stage, respectively. **(D–F)** represent S in V9, V12, and VT stage, respectively. The *X*-axis is the enrichment factor (enriched to the differentially expressed genes in these pathway/background genes in this pathway), the *Y*-axis is the description of the corresponding pathway, the size of bubble represents the number of differentially expressed genes, and the color of bubble represent the *P* value.

To explore the biological and signal transduction pathways associated with DEGs involved in young ear development under heat stress, a KEGG pathway analysis of the DEGs was conducted. After heat stress, DEGs associated mainly with biosynthesis, primary and secondary metabolism, and signal transduction pathways were enriched, and the pathways related to plant hormones were ubiquitous. In the heat-tolerant maize variety, most of the DEGs were clustered around plant hormone signal transduction, zeatin biosynthesis and brassinosteroid biosynthesis pathways (Figures 5A–C). The heat-sensitive maize variety also showed significant expression in pathways associated with plant hormone signal transduction and zeatin biosynthesis (Figures 5D–F). Therefore, we speculate that endogenous plant hormones might play an important role in responses to heat stress.

### Genes Related to Hormones and Heat Stress

**FIGURE 6:**
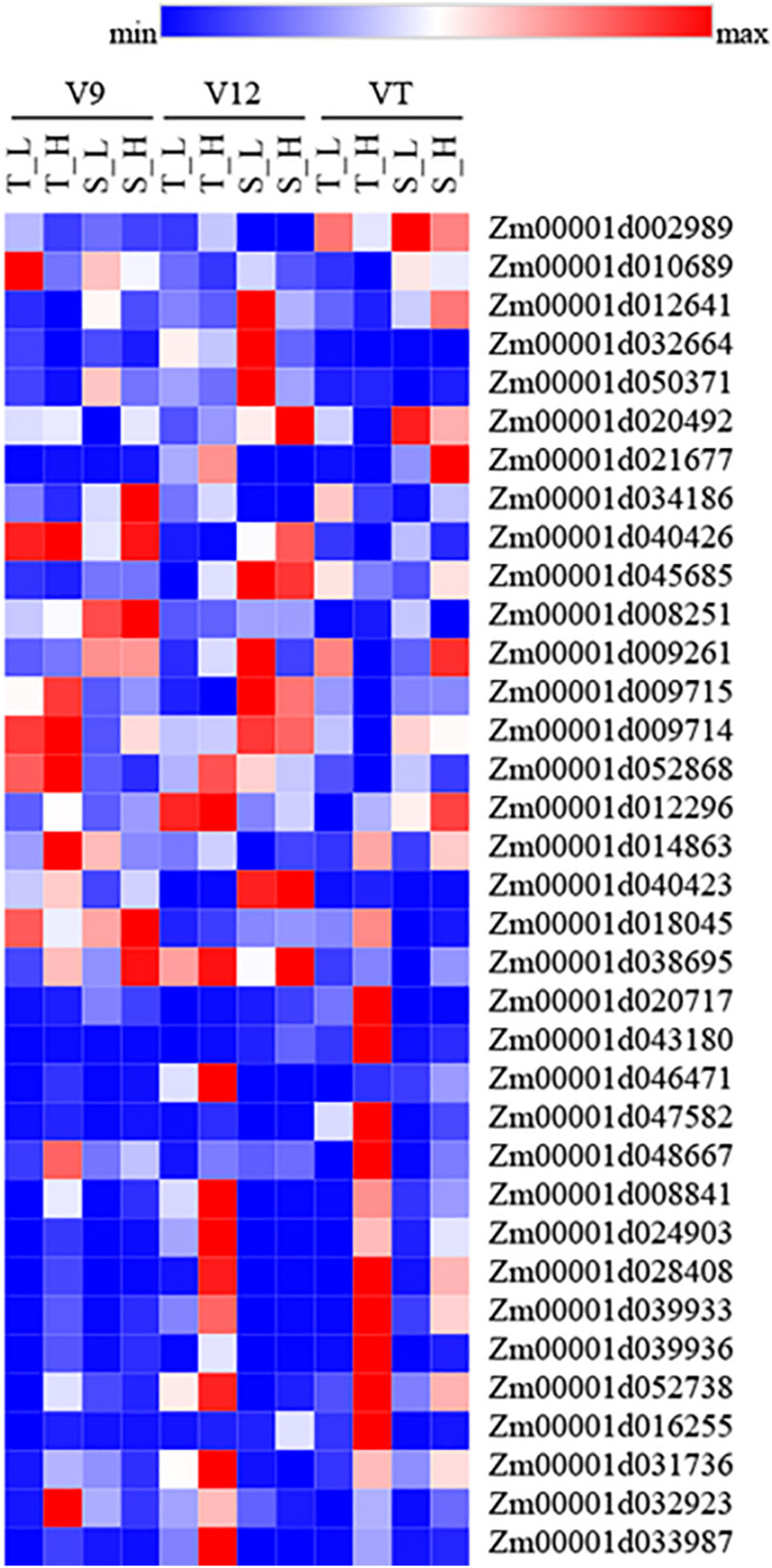
Heatmap of genes which related to hormones and heat shock under heat stress. Red represent up-regulated, and blue represent down-regulated. T and S represent the heat-tolerant variety and the heat-sensitive variety, respectively. L represents the natural environment and H represents the heat-stressed environment. V9, V12, and VT represent the 9^th^ leaf, 12^th^ leaf, and tasseling stages.

We further analyzed the genes associated with hormones and heat shock after heat stress. The results showed that the expression of genes related to ZT biosynthesis (Zm00001d002989, Zm00001d010689, Zm00001d012641, Zm00001d032664, and Zm00001d050371) was down-regulated. The expression of genes related to GA biosynthesis (Zm00001d012296, Zm00001d014863, Zm00001d040423, Zm00001d018045, and Zm00001d038695), ABA (Zm00001d020717, Zm00001d043180, Zm00001d046471, Zm00001d047582, and Zm00001d048667), and heat shock protein (Zm00001d008841, Zm00001d024903, Zm00001d028408, Zm00001d039933, and Zm00001d039936), and heat shock transcription factor (Zm00001d052738, Zm00001d016255, Zm00001d031736, Zm00001d032923, and Zm00001d033987) was up-regulated. The expression of genes in response to SA (Zm00001d020492, Zm00001d021677, Zm00001d034186, Zm00001d040426, and Zm00001d045685) and JA (Zm00001d008251, Zm00001d009261, Zm00001d009715, Zm00001d009714, and Zm00001d052868) was up-regulated in heat-tolerant maize variety; however, the heat-sensitive maize variety showed the opposite trend (Figure 6). There were no significant differences in the gene expression related to auxin and ethylene in each treatment.

### Validation of RNA-seq Data Using qRT-PCR

**FIGURE 7:**
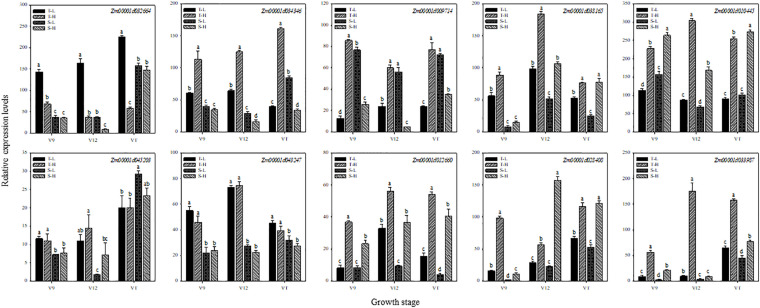
Validation of differential expression of 10 universal genes using qRT-PCR. These genes are related to zeatin (Zm00001d032664), salicylic acid (Zm00001d034346), jasmonic acid (Zm00001d009714), gibberellin (Zm00001d038165), abscisic acid (Zm00001d010445), auxin (Zm00001d045203), ethylene (Zm00001d043247), hormone signal transduction (Zm00001d012660), heat shock protein (Zm00001d028408), and heat shock transcription factor (Zm00001d033987), respectively. T and S represent the heat-tolerant variety and the heat-sensitive variety, respectively. L represents the natural environment and H represents the heat-stressed environment. V9, V12, and VT represent the 9^th^ leaf, 12^th^ leaf, and tasseling stages. Vertical bars represent ± SD of the mean. a, b, c, and d indicate significant difference among different treatments (*n* = 3, *P* < 0.05).

To further verify the expression of genes obtained by RNA-seq, 10 genes related to hormones and heat shock were selected for qRT-PCR. We compared the relative gene expression levels obtained from qRT-PCR with those generated from the RNA-seq analyses related to the 12 treatments. The qRT-PCR results (Figure 7) confirmed that almost all 10 DEGs displayed expression patterns consistent with the expression levels obtained from RNA-seq analyses.

### Endogenous Hormones

**FIGURE 8:**
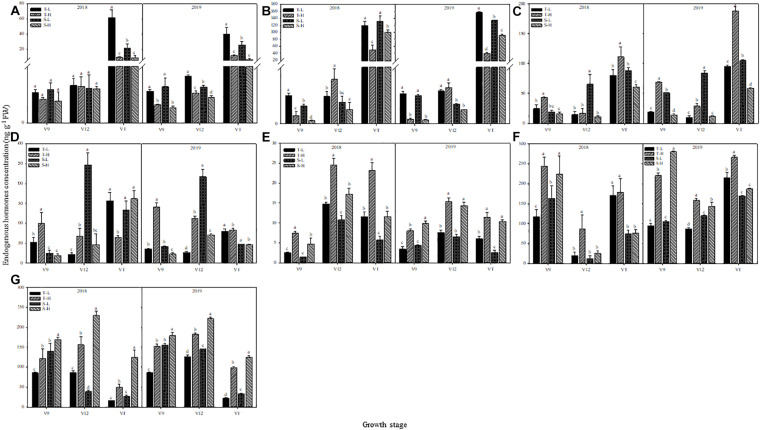
Effect of heat stress on the concentration of zeatin **(A)**, zeatin riboside **(B)**, salicylic acid **(C)**, jasmonic acid **(D)**, gibberellin acid_3_ **(E)**, gibberellin acid_4_ **(F)**, and abscisic acid **(G)** in ears. T and S represent the heat-tolerant variety and the heat-sensitive variety, respectively. L represents the natural environment and H represents the heat-stressed environment. V9, V12, and VT represent the 9^th^ leaf, 12^th^ leaf, and tasseling stages. Vertical bars represent ± SD of the mean. a, b, c, and d indicate significant difference among different treatments (*n* = 3, *P* < 0.05).

The endogenous hormone content showed the same trend in the two-year data and in the detailed analysis based on 2018. The ZT and ZR content decreased after heat stress application, except in the heat-tolerant maize variety during the V12 stage. The largest response in terms of ZT and ZR content was observed in ears that were subjected to heat stress during the VT stage, followed by those subjected to heat stress during the V9 stage. Moreover, after heat stress application during the V9 stage, the decrease in ZT and ZR content for the heat-sensitive maize variety was greater than that for the heat-tolerant maize variety, whereas the opposite results were found after applying heat stress in the V12 or VT stage (Figures 8A,B).

For the heat-tolerant maize variety, the SA content of young ears increased after applying heat stress at any of the experimental stages. The highest increase, which was 75.21% over the control group, occurred after applying heat stress during the V9 stage. In contrast, the content of SA in the heat-sensitive maize variety showed a decreasing trend after heat stress, and the largest decrease, which was 83.69% below the control group, occurred after applying heat stress during the V12 stage. Furthermore, when applying heat stress during the V9 or V12 stages, the SA content in young ears showed greater variations for the heat-tolerant maize variety, but when heat stress was applied during the VT stage, the opposite trend was observed (Figure 8C). The JA content in young ears of the heat-tolerant maize subjected to heat stress during the V9 and V12 stages was 89.17% and 207.04% higher than for the control group, respectively, but it was 65.48% lower than the control group if the heat stress was applied during the VT stage. The heat-sensitive maize variety showed the opposite trend. Despite having different capacities for heat tolerance, both varieties were more sensitive to heat stress application during the V12 stage, and for the heat-tolerant maize variety, the variations were consistently greater than those for the heat-sensitive variety (Figure 8D).

GA_3_, GA_4_, and ABA content in the young ears of both maize varieties increased under heat stress. The greatest increase in GA_3_ content, which was observed after heat stress application during the V9 stage, were 196.54% higher than the control group for the heat-tolerant variety, and 228.00% higher than the control group for the heat-sensitive variety (Figure 8E). In addition, the greatest increase in GA_4_ content, which was observed after applying heat stress during the V12 stage, was 337.64% higher than the control group, for the heat-tolerant variety, and 102.26% higher than the control group, for the heat-sensitive variety (Figure 8F). For ABA content, the greatest increase was 212.63% higher than the control group, and this, occurred in the heat-tolerant maize variety after heat stress application during the VT stage. In the heat-sensitive maize variety, however, the greatest increase, which occurred after the application of heat stress during the V12 stage, was 417.97% higher than the control group (Figure 8G).

In Table 3, the contents of ZT, ZR, JA, and ABA show significant differences between maize varieties, temperatures, and the stages during which heat stress occurred, as well as their interactions. There was no significant difference in SA content between varieties or temperatures, but there was an extremely significant difference between the stages during which heat-stress occurred, as well as in the interactions between various factors. The GA_3_ and GA_4_ content was also extremely significantly affected by the variety, temperature, and stage associated with heat stress occurrence.

**TABLE 3:** ANOVA (*F* value) of the effects of heat stress in different stages on the concentration of endogenous hormones in maize varieties with different capacities for heat tolerance.

Source of variation	Variable						
	ZT	ZR	SA	JA	GA_3_	GA_4_	ABA
Variety	26.32**	82.10**	3.41^ns^	34.47**	187.82**	41.88**	1250.05**
Temperature	124.44**	378.20**	1.47^ns^	86.62**	312.76**	56.75**	4815.57**
Stage	214.95**	3387.49**	237.50**	110.15**	369.05**4	158.51**	1316.52**
Variety × Temperature	31.34**	18.40**	111.86**	63.70**	20.43**4	19.81**	1060.96**
Variety × Stage	27.12**	175.13**	20.16**	118.57**	25.026**	28.61**	77.76**
Temperature × Stage	103.03**	308.33**	16.69**	70.36**	13.45**	4.37*	635.63**
Variety × Temperature × Stage	34.68**	61.63**	4.66*	227.58**	2.31^*ns*^	1.50^ns^	136.96**

ZT, ZR, SA, JA, GA_3_, GA_4_, and ABA represent the zeatin, zeatin riboside, salicylic acid, jasmonic acid, gibberellin acid_3_, gibberellin acid_4_, and abscisic acid, respectively. *Significant at *P* < 0.05 (*n* = 3); **Significant at *P* < 0.01 (*n* = 3); and ^ns^non-significant.

### Correlation Analysis of Plant Endogenous Hormones With Ear Characteristics

Endogenous hormones are an important signaling substance for regulating plant characteristics that facilitate tolerance to external stress. As shown in Table 4, the correlation between ZT and ZR content and the characteristics of young ears after the application of heat stress were consistent. Except for the ZR content of heat-tolerant maize after heat stress application during the V12 stage, the hormone content of plants subjected to any other treatment was positively correlated with the length of young ears, ear diameter, total floret number, and floret fertilization rate, and negatively correlated with the length of the undeveloped part of young ears and the grain abortion rate. The correlations were significant or extremely significant, except for those related to the ZT content of heat-sensitive maize after heat stress application during the V12 stage. The correlations between SA and the observed ear characteristics were similar to those for JA. The SA and JA content of the heat-tolerant variety were negatively correlated with the length of young ears, ear diameter, total floret number, and floret fertilization rate and positively correlated with the grain abortion rate, except for the JA content after heat stress application during the VT stage. For the heat-sensitive maize variety, however, the trend was reversed. Except for the SA content after heat stress application during the V12 stage, the correlation between the hormone level and the ear characteristics all reached significant or extremely significant levels. Similarly, the correlation between the GA_3_ content and the young ear characteristics was similar to that for GA_4_ and ABA. Except for the GA_4_ content of the heat-tolerant maize variety after heat stress application during the VT stage, the GA_3_, GA_4_, and ABA content was negatively correlated with the length of the young ears, ear diameter, total floret number, and floret fertilization rate and positively correlated with the length of the undeveloped part and the grain abortion rate. With respect to the heat-sensitive maize variety, however, the GA_4_ content was not significantly correlated with any ear trait.

**TABLE 4:** Correlation coefficients of endogenous hormones and ear phenotype in maize varieties with different capacities for heat tolerance.

		T						S					
		Length of the young ear	Length of the undeveloped part	Diameter of the young ear	Total number of florets	Floret fertilization rate	Grain abortion rate	Length of the young ear	Length of the undeveloped part	Diameter of the young ear	Total number of florets	Floret fertilization rate	Grain abortion rate
ZT	V9	0.96**	−0.95**	0.95**	0.91*	0.93**	−0.98**	0.91*	−0.86*	0.92**	0.84*	0.89*	−0.87*
	V12	0.92**	−0.94**	0.95**	0.68^ns^	0.93**	−0.93**	0.73^ns^	−0.66^ns^	0.65^ns^	0.42^ns^	0.68^ns^	−0.69^ns^
	VT	0.88*	−0.97**	0.94**	0.85*	0.98**	−0.98**	0.91*	−0.86*	0.86*	0.70^ns^	0.85*	−0.83*
ZR	V9	0.96**	−0.95**	0.97**	0.95**	0.92*	−0.97**	1.00**	−0.91*	0.99**	0.90*	0.98**	−0.99**
	V12	−0.79^ns^	0.89*	−0.80^ns^	−0.62^ns^	−0.84*	0.83*	1.00**	−0.97**	0.98**	0.88*	0.99**	−1.00**
	VT	0.88*	−0.96**	0.88*	0.75^ns^	0.94**	−0.94**	0.60^ns^	−0.76^ns^	0.62^ns^	0.86*	0.73^ns^	−0.76^ns^
SA	V9	−0.91*	0.90*	−0.96**	−0.98**	−0.85*	0.87*	0.92**	−0.80^ns^	0.91*	0.86*	0.91*	−0.88*
	V12	−0.71^ns^	0.80^ns^	−0.70^ns^	−0.57^ns^	−0.75^ns^	0.74^ns^	0.98**	−0.94**	0.94**	0.78^ns^	0.96**	−0.97**
	VT	−0.85*	0.84*	−0.83*	−0.60^ns^	−0.87*	0.88*	0.90*	−0.93**	0.96**	0.84*	0.96**	−0.95**
JA	V9	−0.83*	0.89*	−0.85*	−0.79^ns^	−0.85*	0.85*	0.83*	−0.77^ns^	0.83*	0.65^ns^	0.85*	−0.71^ns^
	V12	−0.99**	0.99**	−0.99**	−0.79^ns^	−0.99**	0.99**	1.00**	−0.97**	0.99**	0.83*	1.00**	−1.00**
	VT	0.86*	−0.96**	0.99**	0.86**	0.97**	−0.97**	−0.95**	0.91*	−0.85*	−0.83*	−0.89*	0.89*
GA_3_	V9	−0.98**	0.99**	−0.98**	−0.95**	−0.94**	0.97**	−0.87*	0.87*	−0.89*	−0.95**	−0.81^*ns*^	0.94**
	V12	−0.97**	0.95**	−0.98**	−0.68^*ns*^	−0.98**	0.98**	−0.94**	0.98**	−0.95**	−0.95**	−0.95**	0.96**
	VT	−0.93**	0.99**	−0.95**	−0.70^*ns*^	−0.97**	0.97**	−0.85*	0.93**	−0.95**	−0.85*	−0.95**	0.95**
GA_4_	V9	−0.99**	0.97**	−0.98**	−0.98**	−0.95**	0.97**	−0.61^ns^	0.77^ns^	−0.62^ns^	−0.82*	−0.50^ns^	0.69^ns^
	V12	−0.95**	0.98**	−0.96**	−0.74^ns^	−0.96**	0.96**	−0.74^ns^	0.70^ns^	−0.72^ns^	−0.46^ns^	−0.78^ns^	0.74^ns^
	VT	0.89*	−0.78^ns^	0.83*	0.94**	0.75^ns^	−0.77^ns^	−0.33^ns^	0.53^ns^	−0.58^ns^	−0.53^ns^	−0.57^ns^	0.59^ns^
ABA	V9	−0.96**	0.95**	−0.95**	−0.91**	−0.90*	1.00**	−0.98**	0.96**	−0.97**	−0.96**	−0.95**	0.99**
	V12	−1.00**	0.99**	−0.99**	−0.78^ns^	−1.00**	−1.00**	−1.00**	0.98**	−0.99**	−0.86*	−1.00**	1.00**
	VT	−0.91*	0.98**	−0.96**	−0.77^ns^	−0.99**	0.99**	−0.96**	0.10**	−0.96**	−0.93**	−1.00**	1.00**

ZT, ZR, SA, JA, GA_3_, GA_4_, and ABA represent the zeatin, zeatin riboside, salicylic acid, jasmonic acid, gibberellin acid_3_, gibberellin acid_4_, and abscisic acid, respectively. T and S represent the heat-tolerant variety and heat-sensitive variety, respectively. L represents the natural environment and H represents the heat-stressed environment. V9, V12, and VT represent the 9^th^ leaf, 12th leaf, and tasseling stages. *Significant at *P* < 0.05 (*n* = 3); **Significant at *P* < 0.01 (*n* = 3); and ^ns^non-significant.

## Discussion

**FIGURE 9:**
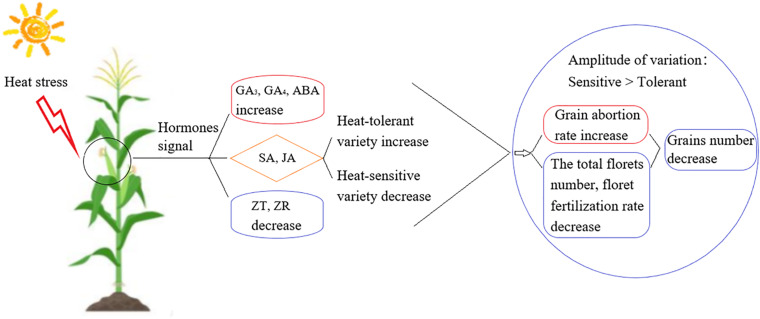
The overview of the difference between heat-tolerant and heat-sensitive maize variety in their heat tolerance.

In this study, we aimed to identify the signaling mechanisms involved in heat stress responses during the development of young ears at three different growth stages for heat-tolerant and heat-sensitive maize varieties (Figure 9). We found that heat stress decreased maize production, even in the heat-tolerant variety, and this will affect maize yields in a warmer world. Deploying increased planting densities may seem like an obvious technique to achieve high maize yields in the future, but there is a decrease in grain number as the density of plantation is increased. Increased planting densities also lead to canopy closure and a reduced tolerance to adversity (Xu et al., 2017), especially to heat stress (Cicchino et al., 2010; Ordóñez et al., 2015). The number of grains is the most significant factor affecting yield, and thus, reducing the heat-stress-induced reduction in grain numbers is the key to obtaining high and stable yield (Prasad et al., 2011). The grain number depends on the total number of florets, number of fertilized florets, and effective number of grains that develop from a fertilized floret (Dong et al., 2010). In plants under heat stress, the floret abortion rate, pollen viability, and ability to germinate are decreased; moreover, the silking time is delayed and filament viability is decreased. As a result, the pollination and fertilization processes are blocked, which decreases the fertilization rate, increases the abortion rate, and ultimately reduces the grain number (Dreccer et al., 2014; Prasad and Djanaguiraman, 2014). The spikelet and floret differentiation stages are key stages in the young ear differentiation process. During these stages, the ear length, ear diameter, floret number and grain abortion rate are the key factors that determine the grain number (Mohammed and Tarpley, 2011). Our results showed that when heat stress occurred during the V9, V12, or VT stage, the length of the young ear was 8.88–75.97% shorter than in the control group and the ear diameter was 23.63–74.44% smaller. The greatest impact occurred in plants subjected to heat stress during the V9 stage. At this stage, the ear is in the growth cone extension phase, and heat stress significantly inhibits the longitudinal and transverse elongation of the ear. At the same time, it renders the top of the ear dysplastic, thus suppressing its capability to form normal florets. Moreover, heat stress causes the growth stage to be delayed, the filaments to appear later, the florescence to be insufficient, and the fertilization process of filaments to be seriously impaired, especially for those filaments that grow at the top and the base of the ear. Finally, an increased grain abortion rate ultimately results in a significant reduction in the number of grains. This effect is more obvious for the heat-sensitive maize variety than for the heat-tolerant variety, indicating that the young ear differentiation process is sensitive to heat stress.

About 5% of the genes in the plants were up-regulated more than twice after the plants were subjected to heat stress (Finka et al., 2011). In the current study, the transcriptome analysis revealed that in the heat-tolerant maize variety, the DEGs that were induced by heat stress accounted for 0.64–3.02% of the total number of expressed genes, and that most genes were down-regulated. In the heat-sensitive maize variety, the DEGs that were induced by heat stress accounted for 0.12–4.10% of the total number of expressed genes, and most genes were up-regulated. Evidently, maize varieties with different capacities for heat tolerance exhibit different regulation patterns for gene expression when subjected to heat stress. When plants were under heat stress, the expression of many heat-resistant genes changed, such as those encoding hormones, heat shock proteins, and heat shock transcription factor, among others (Liu and Chen, 2015). Interestingly, although there were complicated interactions between different genes, GA might induce a gene associated with heat-tolerance (Ko et al., 2007). It is reported that ABA and SA are also connected with heat signal transduction under heat stress (Kotak et al., 2007). Furthermore, heat shock proteins, can be induced as a stress protein by many adversities, including temperature, water, and saltinity, which could alleviate the damage caused by stress and repair the plant in the meantime (Grigorova et al., 2011). Transcriptome analyses have shown that different crops exhibit different pathways in their response to heat stress; moreover, they have shown that most of these pathways are hormone signal transduction pathways (Larkindale et al., 2005; Qin et al., 2008). This observation indicates that, as important signaling molecules in response to heat stress, hormones play an irreplaceable role in the perception and conduction of heat signals. According to the results of our study, the enriched DEGs were related to pathways associated with hormone biosynthesis and signal transduction. The genes related to ZT were down-regulated after heat stress, while genes related to GA, ABA, heat shock proteins and heat shock transcription factor were up-regulated. Gene related to SA and JA were up-regulated in the heat-tolerant maize variety but down-regulated in the heat-sensitive maize variety. The qRT-PCR data supported the data obtained from RNA-seq, proving that our approach of finding the differences between endogenous hormone levels in ears subjected to heat stress is a valid approach for exploring the response of maize varieties with different heat tolerance to heat stress.

Endogenous plant hormones are closely related to plant growth, development, and yield formation, and their existence and relative content can be used as an indicator of the physiological state of a plant, particularly as heat stress has the greatest influence on a plant’s hormonal state (Scalabrin et al., 2016). Abiotic stress usually causes an increase in ABA content, which is one of the fastest ways to obtain heat tolerance (Yamaguchi-Shinozaki and Shinozaki, 2006; Raghavendra et al., 2010). In sweet maize, the expression of genes related to ZT biosynthesis is down-regulated in heat-tolerant variety under heat stress, so as to maintain a low growth level (Shi et al., 2017). Similarly, in grains, heat stress causes the ZT and ZR content to decrease in maize, rice, and wheat, but it causes the ABA and GA_3_ content to increase (Sobol et al., 2014; Yang et al., 2014; Wu et al., 2016). Spraying ABA and GA also significantly increases grain weight and thermotolerance (Yang et al., 2011; Mo et al., 2018). Our results showed that the ZT and ZR content of maize ears decreased under heat stress and that the decrease in the heat-tolerant maize variety was greater when heat stress occurred in the V12 or VT stage. The GA_3_, GA_4_, and ABA content of young ears increased under heat stress, and the increase in ABA was greater for the heat-sensitive maize variety. The changes in the ZT, ZR, GA_3_, GA_4_, and ABA content of young ears under heat stress were significantly or extremely significantly correlated to the length of the young ear, ear diameter, length of the undeveloped part at the top of the ear, total floret number, floret fertilization rate, and grain abortion rate. The results showed that these hormones were all involved in the heat stress response of young ears and that they had a significant adverse effect on their development. As signaling substances in response to biological and abiotic stresses (Clarke et al., 2009), SA and JA play key roles in the establishment of thermotolerance (Zhou et al., 2010). The SA content increases rapidly during the first 30 min of heat stress application and subsequently decreases sharply, indicating that SA can quickly transmit heat signals. Moreover, the SA content in the leaves of heat-tolerant maize increases under heat stress, while it decreases in heat-sensitive maize (Dinler et al., 2014). The results from our study showed that the SA and JA content of young ears in the heat-tolerant maize variety increased under heat stress and that the trend was reversed for ears of the heat-sensitive maize variety. Moreover, the hormone content of ears of the heat-sensitive maize variety were positively correlated with the total floret number and the floret fertilization rate and negatively correlated with the grain abortion rate. Therefore, the decreases in SA and JA content under heat stress might be an important reason for the low thermotolerance and poor development of young ears of the heat-sensitive maize variety. Taken together, heat stress caused changes in the hormone levels in young ears, and inhibited their development, which was the main reason for the decrease in grain numbers under heat stress. Nevertheless, the content of both GA and ABA increased in maize ear under heat stress, although they are antagonistic hormones (Yang et al., 2017). In addition, a similar GA and ABA increase under temperature or water stress has been described in wheat, rice and Arabidopsis (Van et al., 2009; Yang et al., 2014; Wu H. et al., 2019). Therefore, a set of complex physiological reaction mechanisms must exist, which we will take exploring them as the focus of our further research.

## Conclusion

Transcriptome analysis, of a heat-sensitive and a heat-tolerant maize variety subjected to heat stress, showed that the DEGs in the heat-sensitive maize variety were up-regulated rather than down-regulate under heat stress, and that the heat-tolerant maize variety had the opposite response. A KEGG analysis showed that the DEGs were mostly enriched in pathways associated with hormone signaling in response to heat stress. After applying heat stress, the ZT and ZR content decreased, and the GA_3_, GA_4_, and ABA content increased, which resulted in the floret number, floret fertilization rate, and grain number decreasing and the grain abortion rate increasing. Furthermore, there was evidence that heat- stress-induced increases in SA and JA content were the key reasons for high thermotolerance of the heat-tolerant maize variety.

## Data Availability Statement

All sequences were deposited in the National Center for Biotechnology Information (NCBI) and can be accessed in the Short Read Archive (SRA) under accession number: SRP250388.

## Author Contributions

PL and H-QW initiated and designed the research. H-QW analyzed the data, and wrote the manuscript. J-WZ, BZ, and B-ZR provided advice on the experiments. All authors contributed to the article and approved the submitted version.

## Conflict of Interest

The authors declare that the research was conducted in the absence of any commercial or financial relationships that could be construed as a potential conflict of interest.

## Supplementary Material

Supplementary Figure 1 Diurnal changes of mean temperature **(A)** and mean relative humidity **(B)** in heat-stressed and natural environments. The heat-stressed and natural environments represent the temperature or relative humidity inside and outside the sheds. The hourly value is the average of 10 days’ measurements.

Supplementary Table 1 Primer design for quantitative real-time PCR (qRT-PCR) of commonly expressed genes from RNA sequencing data.
