# Supplementary figures and images for: Endogenous Hormones Inhibit Differentiation of Young Ears in Maize (Zea mays L.) Under Heat Stress
Source: Front Plant Sci. 2020 Oct 22;11:533046. doi: 10.3389/fpls.2020.533046 (PMC7642522; doi:10.3389/fpls.2020.533046)

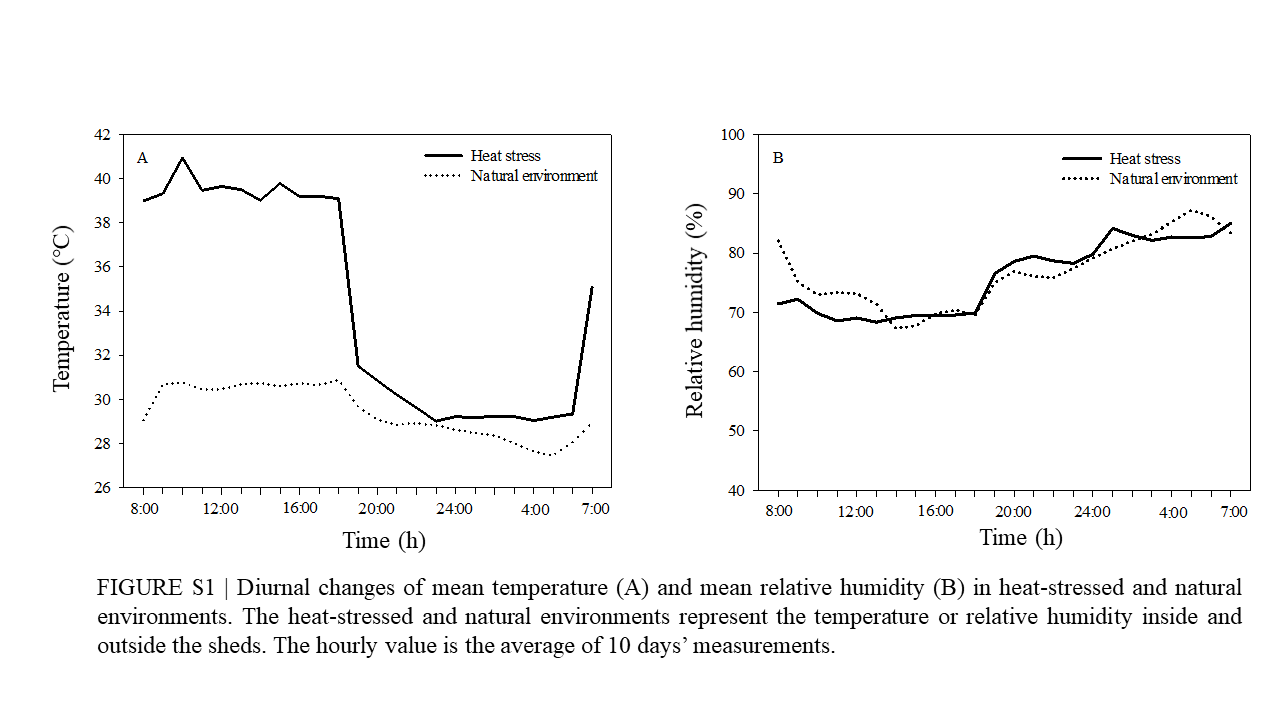

Supplement: Supplementary Figure 1 — Diurnal changes of mean temperature (A) and mean relative humidity (B) in heat-stressed and natural environments. The heat-stressed and natural environments represent the temperature or relative humidity inside and outside the sheds. The hourly value is the average of 10 days’ measurements. [file Figure_1.TIF]
