# Supplementary material for: Endogenous Hormones Inhibit Differentiation of Young Ears in Maize (Zea mays L.) Under Heat Stress
Source: Front Plant Sci. 2020 Oct 22;11:533046. doi: 10.3389/fpls.2020.533046 (PMC7642522; doi:10.3389/fpls.2020.533046)
Supplement: Supplementary Table 1 — Primer design for quantitative real-time PCR (qRT-PCR) of commonly expressed genes from RNA sequencing data. [file Table_1.docx]

TABLE S1 | Primer design for quantitative real-time PCR (qRT-PCR) of commonly expressed genes from RNA sequencing data

| Gene name | Forward (5–3´) | Reverse (5–3´) | Annealing Temperature (℃) | Product  Length (bp) |
| --- | --- | --- | --- | --- |
| Zm00001d038165 | CTTTGCCCCGAGGAGATCG | AAGTTGGAGATGAGCACCCA | 60℃ | 250 |
| Zm00001d010445 | GACGACGACCTGCACATACT | GAACGACTCGATCACCAGGG | 60℃ | 132 |
| Zm00001d034346 | AAGCGCGGGATTGATATGGT | TGAGGAGCAAAGACCTGAGC | 60℃ | 110 |
| Zm00001d012660 | GGAATCCTATGGCGGTGGAA | GACCTTGGCTTCAGCAGGAA | 60℃ | 128 |
| Zm00001d009714 | AACATTTGAGGAGGTTTGGGA | ATTGGGCTTGAGGATTTTTGTC | 60℃ | 220 |
| Zm00001d032664 | AGGGCCTCATCCTCATCTACC | ACGTACATCACCGGGTCGTC | 60℃ | 101 |
| Zm00001d028408 | GCCGGATGAGTGCGACAA | CCCAACCCACGCTAGTGCTAC | 60℃ | 135 |
| Zm00001d033987 | TCTCTGCTCTGGTCCTTCAC | CCAAACGAACGAACGAACGAA | 60℃ | 243 |
| Zm00001d045203 | CTGCATCGTCCTTACGTGGT | TTGAGCCGCTTCACTGACTT | 60℃ | 107 |
| Zm00001d043247 | AAAGTGGAGGAAACCGAGGG | ACACCCAGCTTGATGACGAA | 60℃ | 148 |
| Zea mays-GAPDH | TATCATGGGTTATGTGGAAGAGGA | TGACGAAGTGGTCGTTCAGAG | 60℃ | 110 |
